# NBDHEX re‐sensitizes adriamycin‐resistant breast cancer by inhibiting glutathione S‐transferase pi

**DOI:** 10.1002/cam4.5370

**Published:** 2022-10-20

**Authors:** Huanhuan Sha, Renrui Zou, Ya Lu, Yujie Gan, Rong Ma, Jifeng Feng, Dan Chen

**Affiliations:** ^1^ Department of Chemotherapy Jiangsu Cancer Hospital, The Affiliated Cancer Hospital of Nanjing Medical University, Jiangsu Institute of Cancer Research Nanjing Jiangsu China; ^2^ The Fourth Clinical School of The Affiliated Cancer Hospital of Nanjing Medical University, Jiangsu Cancer Hospital, Jiangsu Institute of Cancer Research Nanjing Jiangsu China; ^3^ Research Center of Clinical Oncology, Jiangsu Cancer Hospital, The Affiliated Cancer Hospital of Nanjing Medical University, Jiangsu Institute of Cancer Research Nanjing Jiangsu China

**Keywords:** adriamycin, breast cancer, GSTpi, NBDHEX, resistant

## Abstract

**Purpose:**

Adriamycin is a novel chemotherapeutic agent of great benefit for treating breast cancer. However, adriamycin ‐resistance remains a major obstacle. The vital Glutathione transferase P1 (GSTPi) inhibitor 6‐(7‐nitro‐2,1,3‐benzoxadiazol‐4‐ylthio) hexanol (NBDHEX) has recently shown antitumor activity in various cancers. In this study, we analyzed the effect of NBDHEX and adriamycin combination against breast cancer in vitro and in vivo.

**Methods:**

CCK‐8 assay was performed to test cell viability. The location and expression level of GSTpi was determined by immunofluorescence and Western blot in cells and immunohistochemistry staining in tissues. The enzyme activity test was applied to detect the effect of NBDHEX on the activity of GSTpi. The apoptosis related proteins' expression was tested using Western blot. The phosphorylation sites of GSTpi were detected by mass spectrometry. Antitumor effects of single treatment or co‐administration of adriamycin and NBDHEX were evaluated in nude mice.

**Results:**

NBDHEX treatment inhibited GSTpi enzyme activity and co‐administration of adriamycin and NBDHEX promoted apoptosis of adriamycin‐resistance breast cancer cell. Moreover, drug combination of NBDHEX and adriamycin significantly enhanced tumor growth inhibition compared with single agent.

**Conclusion:**

NBDHEX serves as a good candidate for combination with adriamycin, offering new insights for breast cancer treatment.

## Graphical abstract

This article indicates that NBDHEX represents a good candidate for combination therapies with adriamycin, offering new perspectives for the treatment of breast cancer.

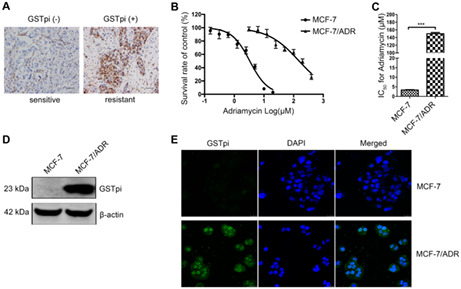

## 1 INTRODUCTION

Breast cancer is the most common cancer in women, which accounts for about 24.5% all new cancer diagnoses in women. Additionally, it remains the leading cause of death among females worldwide.^1^ Adriamycin (ADR) is a novel chemotherapeutic agent of great benefit for breast cancer treatment. However, ADR‐resistance remains a major obstacle. Multiple mechanisms of ADR resistance in cancers have been proposed in the last decades, including decreased drug accumulation, upregulating expression of drug efflux transporters such as multidrug resistance protein and P‐ glycoprotein, increased DNA repair activity, or excessive detoxification by Glutathione S‐transferases (GSTs)/ Glutathione (GSH) systems.^2^, ^3^, ^4^

GSTs is a family of enzymes involved in xenobiotic detoxification via the conjugation of reduced GSH with a wide variety of hydrophilic molecules. Overexpression of GSTs in human cancer cells catalyze the conjugation of anticancer drugs to GSH. Therefore, drugs can be efficiently extruded from cells by export pumps, which plays an important role in cancer susceptibility to anticancer drugs.^5^, ^6^, ^7^, ^8^ The dominant member of GSTs is the GSTpi isoenzyme, encoded by GSTP1 gene which is located on chromosome 11q13.^9^ Apart from its xenobiotic detoxification function, GSTpi is also involved in cell proliferation, apoptosis and other biological processes by regulating phosphorylation of key signaling effectors, such as C‐jun NH2‐terminal kinase (JNK) and TNF receptor‐associated factor 2 (TRAF2).^10^, ^11^ Our previous study also found GSTpi could inhibit the phosphorylation and nuclear translocation of signal transducer and activator of transcription 3, thereby affecting cell proliferation and migration.^12^ However, little attention has been paid to GSTpi's own phosphorylation so far.

Extensive efforts have been dedicated to develop compounds which may modulate GSTpi's biological activity considering its significant functions. However, inhibitors such as GSH derivatives and ethacrynic acid are often actively removed from cell by export pumps and both of these two kinds inhibitors lack affinity or class specificity.^13^, ^14^ Fortunately, nitrobenzoxadiazole (NBD) derivatives are synthesized and evaluated as efficient inhibitors.^15^, ^16^, ^17^ Among them, NBDHEX has emerged as an excellent inhibitor of GSTpi at micromolar or submicromolar recently. It can disrupt the interaction between the GSTpi‐ TRAF2 and GSTpi‐ JNK to induce apoptosis, and avoid extrusion from cancer cells by export pumps.^10^, ^11^, ^18^, ^19^ Additionally, a recent study demonstrates that NBDHEX acts as late‐phase autophagy inhibitor, opening its new therapeutic opportunities.^20^ We summarized its satisfactory anticancer ability in various animal models and cancer cell lines previously, including acute myeloid leukemia, small cell lung cancer, osteosarcoma, and so on.^21^ However, the role of NBDHEX on GSTpi in ADR‐resistance breast cancer remains unexplored.

Here, we performed experiments to evaluate the effect of NBDHEX on ADR‐resistance breast cancer. NBDHEX inhibited GSTpi enzyme activity and promoted apoptosis of ADR‐resistance breast cancer cell. Furthermore, GSTpi phosphorylation might be correlated with enzyme activity and its nuclear translocation. Drug combination of NBDHEX and adriamycin also significantly enhanced tumor growth inhibition. We demonstrated that NBDHEX re‐sensitized ADR‐resistant breast cancer cell to adriamycin by inhibiting GSTpi.

## 2 MATERIALS AND METHODS

### 2.1 Cell culture and transfection

Human breast cancer cell line MCF‐7 was purchased from the Cell Bank of the Chinese Academy of Sciences. Identities of cell line was authenticated using DNA profiling (short tandem repeat). Adriamycin (ADR)‐resistant cell line was established by exposing the MCF‐7 to increasing concentrations of ADR step‐by‐ step. MCF‐7/ADR and MCF‐7 were grown in Dulbecco's modified Eagle's medium (DMEM) (Thermo Fisher Scientific) supplemented with 10% fetal bovine serum (Thermo Fisher Scientific), 0.08 mg/ml streptomycin and 80 U/ml penicillin (KeyGEN BioTECH). Cells were maintained at 37°C with 5% CO2 atmosphere. For cell transfection experiment, cells at approximately 80% confluence were collected and seeded into indicated plates. Lipofectamine 3000 (Thermo Fisher Scientific) was applied to perform transient transfection referring to the manufacturer's instructions.

### 2.2 Drugs

ADR (10 mg) was obtained from Hisun Phamacrutical Co., Ltd. NBDHEX (20 mM) stock solutions were kindly provided by Dr Luolan from State Key Laboratory of Pharmaceutical Biotechnology, School of Life Sciences, Nanjing University. NBDHEX was dissolved to the appropriate concentration just before use and final DMSO (Amresco) concentration was less than 0.1%. Both ADR and NBDHEX stock solutions were diluted in DMEM to prepare working concentrations.

### 2.3 Reagents, plasmids and protein purification

pcDNA3, pcDNA3‐Flag‐GSTpi (WT), pLKO.1, Pet28a‐GSTpi (WT) were kept in our laboratory. Plasmid including pcDNA3‐Flag‐GSTpi (Y7F), pLKO.1‐GSTpi shRNA, pLentiCRISPRv2‐GSTpi were constructed and Endofree Plasmid Preparation kit (Qiagen) was applied to purify plasmids. Antibodies including anti‐PARP, β‐actin, GAPDH and FLAG were obtained from Cell Signaling Technology Inc. Anti‐GSTpi antibody was purchased from BD Bioscience and from Abcam. Lamin B2 antibody was obtained from Proteintech. A/G beads were from Santa Cruz Biotechnology. The IRdye 680 conjugated IgG secondary antibodies were purchased from LI‐COR Biosciences. Secondary antibody in immunofluorescence was from Thermo Fisher Scientific. Proteins including GSTpi (WT), GSTpi (Y3F), GSTpi (Y7F), GSTpi (Y63F), GSTpi (Y198F) were expressed in Escherichia coli and purified as follows: 500 ml cell precipitation was suspended with 80 ml Ni‐IDA Binding‐Buffer (20 mM Tris–HCl, 0.5 M NaCl, 20 mM imidazole, pH 8.0). The supernatant was obtained after ultrasonic fragmentation (power 200 W, working 5 sec, intermittent 7 sec, total 25 min) and centrifugation at 12000 g for 20 min. Then the supernatant was rinsed with Ni‐IDA Binding‐Buffer at a speed of 0.5 ml/min followed by Ni‐IDA Washing‐Buffer (50 mM imidazole, 20 mM Tris–HCl, 0.5 M NaCl, pH 8.0) at 1 ml/min rate until the OD_280_ value of effluent fluid reached the baseline. Finally, proteins were eluted by Ni‐IDA Elution‐Buffer (250 mM imidazole, 20 mM Tris–HCl, 0.5 M NaCl, pH 8.0) at 1 ml/min rate and the effluent was collected for analysis.

### 2.4 Cell viability test

Cell Counting Kit‐8 (CCK‐8) assay (Dojindo) was used to evaluate cell viability at different drug concentrations. Cells were seeded into 96‐well plates at a density of 8 × 10^3^/well and different concentrations of ADR, NBDHEX, or ADR + NBDHEX were added to the cells (quadruplicate wells per condition). Two days later, 10 μL of CCK‐8 solution was diluted at a ratio of 1:10 and 100 μL diluted CCK‐8 was added to each well. The absorbance at 450 nm was measured by SpectraMax (Molecular Devices) after incubation for another 4 h. Drug dose–response curves were generated and the half maximal inhibitory concentration (IC50) was calculated for drug sensitivities.

### 2.5 Immunofluorescence

Prepared cells grown on culture dishes (NEST Biotechnology) were fixed in 4% paraformaldehyde for 30 min, permeabilized in 0.5% Triton X‐100 (Amresco) for 20 min and blocked in 5% bovine serum albumin (BSA) (Thermo Fisher Scientific) for 1 h. Then, cells were incubated in blocking buffer containing anti‐GSTpi (1:200) overnight. After three phosphate‐buffered saline (PBS) (KeyGEN BioTECH) washing, cells were treated with blocking buffer containing secondary fluorescence‐antibody (1:1000) for 1 h. Subsequently, 0.1 μg/ml DAPI (Thermo Fisher Scientific) was used to stain cell nuclei after PBS washing. Immunofluorescence were imaged using the fluorescence microscope.

### 2.6 Western blotting analysis

Cells were collected in phosphate buffered solution (PBS) and washed twice with cold PBS by centrifuging at 300 × g for 3 min. All procedures were carried out at 4°C unless otherwise mentioned. Total proteins were extracted using RIPA lysis buffer (Beyotime Biotechnology). Nuclear and cytoplasmic extraction kit from KeyGEN BioTECH Corp.Ltd., was used to harvest cytosolic and nuclear proteins, according to the manufacturer's instructions. Then, bicinchoninic acid protein assay (Beyotime Biotechnology) was applied to measure protein concentrations. Equal amounts of prepared proteins were loaded on 10%–12% sodium dodecyl sulfate polyacrylamide gels. Subsequently, proteins were transferred onto polyvinylidene difluoride membranes (Merck Millipore), which were then blocked in 5% skim milk for 2 h. Then membranes were probed with primary antibodies including PARP (1:1000), GSTpi (1:1000), FLAG (1:1000), GAPDH (1:1000), β‐actin (1:1000) and Lamin B2 (1:1000). After incubation overnight, IRdye 680‐labeled IgG secondary antibodies (1:5000) were incubated for 1 h. The Odyssey infrared imaging system (LI‐COR Biosciences) was applied for protiens visualization. β‐actin, GAPDH and Lamin B2 were utilized as internal controls, respectively.

### 2.7 Enzyme activity

GST activity assay kit (KeyGEN BioTECH) was used to measure GST activity according to the manufacturer's instructions. Cancer cell lines were collected by centrifugation at 800 g for 5 minutes at 4°C and washed twice in PBS. Cells were suspended in PBS, lysated by a 10‐second sonication, and then centrifuged at 13,000 g for 10 minutes at 4°C. Finally, aliquots of the supernatant were used to measure the GST activity which was determined spectrophotometrically at 412 nm. The amount of enzyme that was able to reduced 1 μM of GSH per minute at 37°C was defined as one unit of GST activity.

### 2.8 Mass spectrometry analysis

Proteins were immunoprecipitated using anti‐GSTpi antibody at 4°C overnight followed by A/G–agarose beads incubation for 2 h. Then, the complex was washed four times with the lysis buffer and eluted from beads, and finally the GSTpi complex was analyzed by mass spectrometry as we previously described.^12^

### 2.9 Immunohistochemistry assay

Immunohistochemistry (IHC) assay was performed on already available formalin‐fixed, paraffin‐embedded tissues sections, which were obtained from three breast cancer patients sensitive and three patients resistant to ADR treatment in Nanjing Medical University Affiliated Cancer Hospital from September 2016 to August 2017. Sections were incubated with anti‐GSTpi (Proteintech) at a dilution of 1:100 overnight at 4°C followed by rinsing 2 × 5 min TBS 0.025% Triton. Then, they were incubated with secondary antibody diluted in buffer according to the manufacturer's instructions for 30 min. Detection was performed by the pathologist who was blinded to the clinical treatment information.

### 2.10 Animal studies

Four to six weeks old male BALB/c nude mice were purchased from Shanghai experimental animal center, China Academy of Science (Shanghai, China) and maintained in the animal center of Nanjing Medical University. Mice were housed in controlled temperature (22 ± 2°C) under 12 h light/dark cycles and relative humidity (50%–60%). A total of 200 μL MCF‐7/ADR cells suspensions (3 × 10^7^ cells per ml) were inoculated subcutaneously after 1‐ week acclimatization. Treatment started when tumor nodules were palpable. Mice were randomly divided into four groups (5 per group). NBDHEX was given at the dose of 0.5 mg/kg/d, while ADR was administered intraperitoneally at the dose of 5 mg/kg/d. Drug effects on mice were evaluated by using single agent or combination of ADR and NBDHEX. Animal body weight was monitored every 3 days. The tumors were collected and measured immediately when the experiment ended. For HE staining, isolated tissues were embedded in paraffin and processed for staining according to routine protocols. Briefly, 5 μm longitudinal sections were stained with hematoxylin solution for 5 min after deparaffinization and rehydration. Then, the sections were dipped in 1% acid ethanol (1% HCl in 70% ethanol), rinsed in distilled water and stained witheosin solution for 3 min. At last, they were cleared in xylene followed by dehydration with graded alcohol. The slides were then photographed and analyzed under an optical microscope.

### 2.11 Statistical analysis

Experiments were carried out in triplicate independently. Statistical analysis was performed using GraphPad Prism 6.0 and data were presented as mean ± SD. Statistical evaluation was done using the Student's *t* test or analysis of variance (ANOVA). The criterion for statistical significance used was *p* < 0.05.

## 3 RESULTS

### 3.1 GSTPi location and expression in ADR‐sensitive or resistant breast cancer tissues and cell lines

**FIGURE 1:**
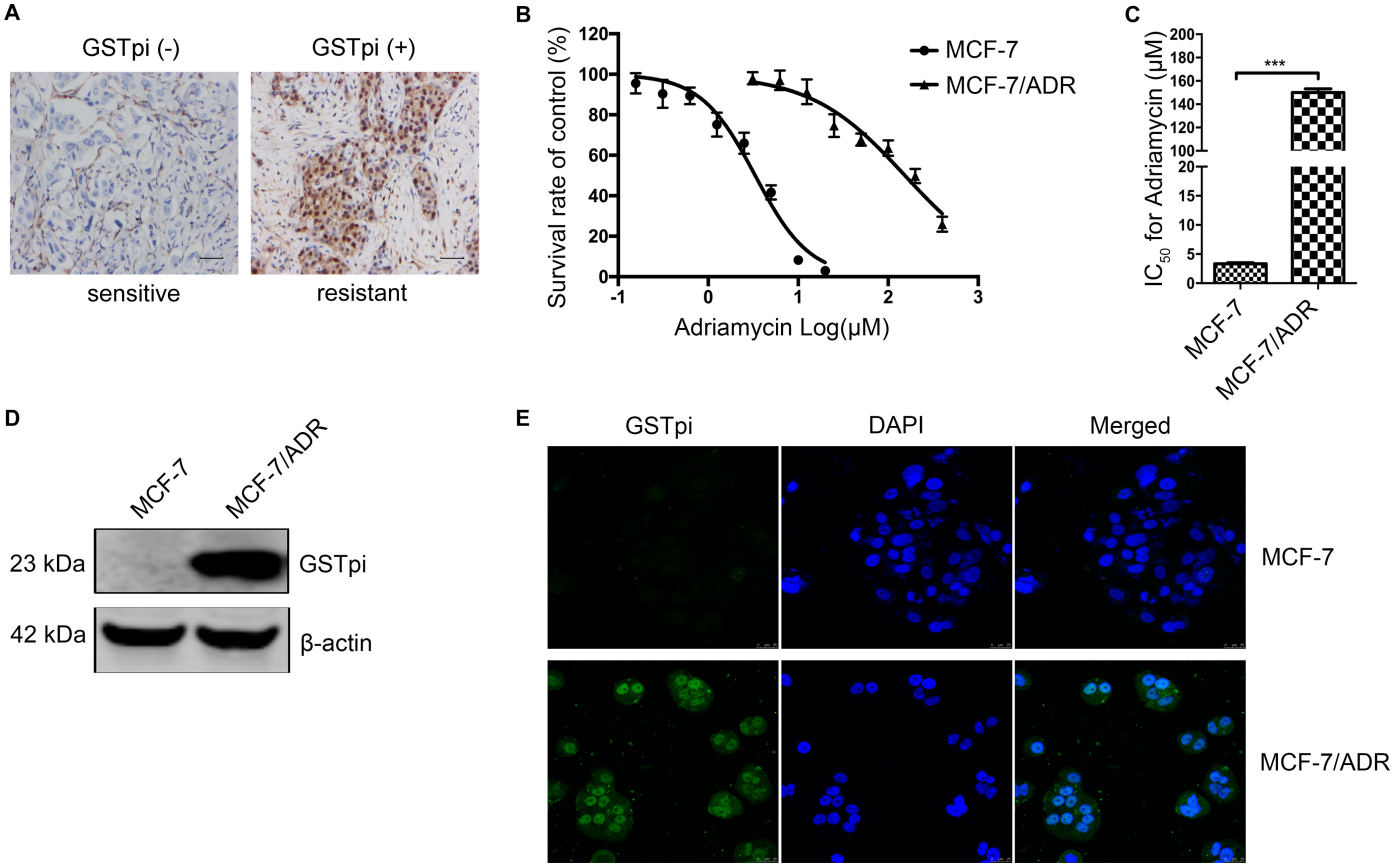
GSTPi expression and location in ADR‐sensitive or resistant breast cancer tissues and cell lines. (A) GSTpi expression and location in ADR‐sensitive or resistant breast cancer tissues. Scale bars represent 50 μm. (B) The adriamycin IC50 values of MCF‐7 and MCF‐7/ADR. (C) The fold increase of adriamycsin resistance in MCF‐7/ADR compared with MCF‐7. (D) Western blot analysis of GSTpi protein in MCF‐7 and MCF‐7/ADR. (E) The immunofluorescence scanning of GSTpi in MCF‐7 and MCF‐7/ADR. Scale bars represent 25 μm. **p < 0.001.

We examined the GSTpi expression in both ADR‐sensitive or resistant breast cancer tissues. IHC staining showed that the expression level of GSTpi in ADR‐resistant tissues was higher than that in ADR‐sensitive tissues. Besides, GSTpi distribution in the nucleus of ADR‐resistant tissues was observed (Figure 1A). ADR‐resistant variant of MCF‐7 was obtained via exposing cell to 3.4 μM ADR until the cells achieved similar growth rates to untreated cells. Then, selection was performed by raising ADR concentration step‐by‐step. After 6 months, MCF‐7/ADR was established and maintained in culture for a steady concentration. The ADR IC50 values and the fold increase of resistance in MCF‐7/ADR were showed in Figure 1. From the results, the IC50 values of MCF‐7 and MCF‐7/ADR were 3.35 ± 0.18 μM and 150.57 ± 3.07 μM respectively (Figure 1B). Compared with the parental cell line MCF‐7, the MCF‐7/ADR cell line were 44.8 folds more resistance to ADR (Figure 1C). To evaluate the GSTpi expression in MCF‐7/ADR, MCF‐7 cell line was used as a negative control. From Figure 1D, we found that MCF‐7/ADR had higher GSTpi expression level than the parental cell line. Similar evidence was found with the immunofluorescence result (Figure 1E). These findings indicated that, increased resistance to ADR might be associated with enhanced GSTPi protein level, as reported in other human cancer cell lines.^22^

### 3.2 GSTPi expression level partially changed ADR‐resistance in breast cancer

**FIGURE 2:**
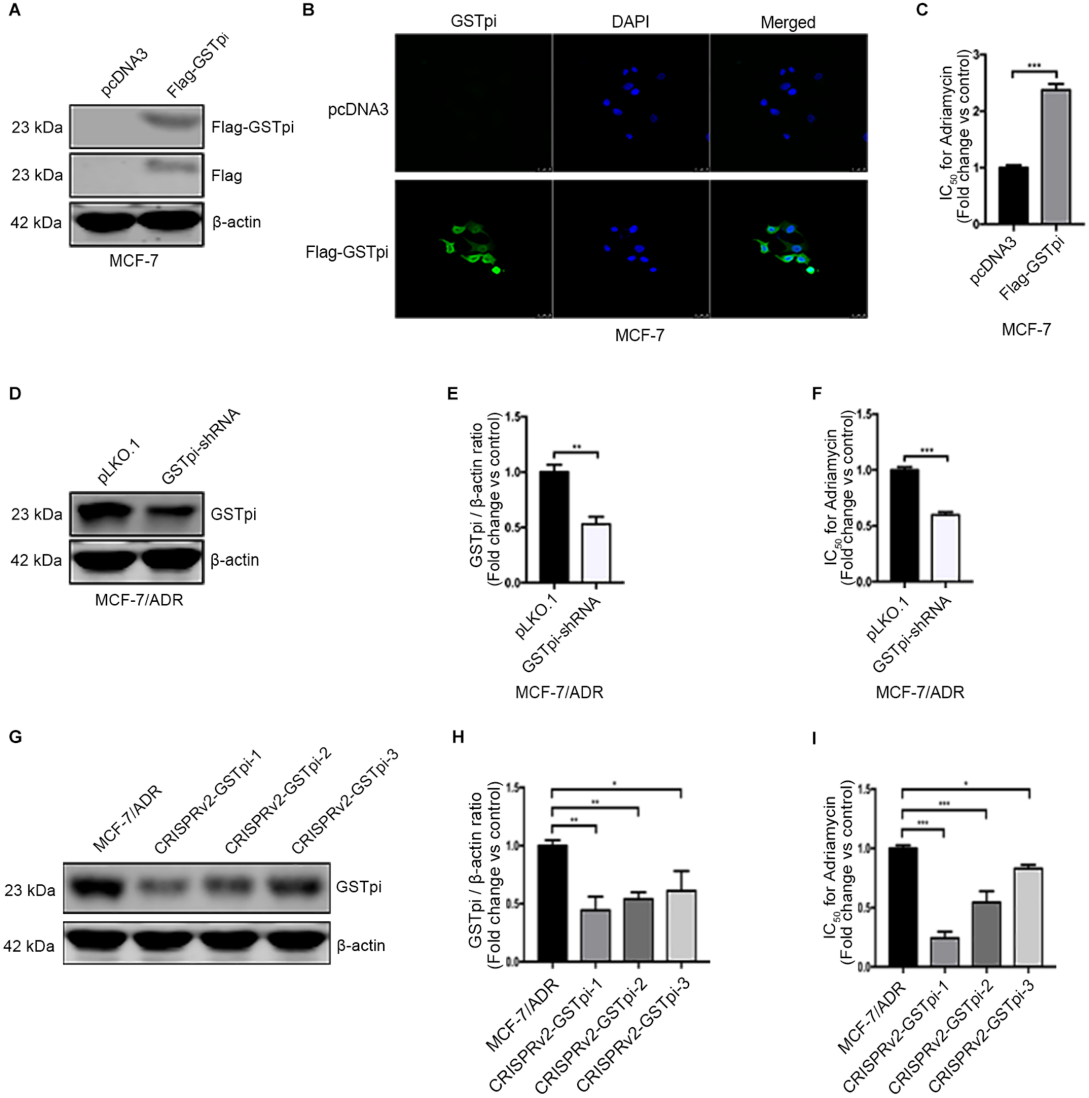
Adriamycin resistance was related to the expression of GSTpi. (A) Western blot analysis of GSTpi protein in MCF‐7 after transfected with empty vector or pcDNA3‐Flag‐GSTpi. (B) The immunofluorescence scanning of GSTpi in MCF‐7 after transfected with empty vector or pcDNA3‐Flag‐GSTpi. Scale bars represent 25 μm. (C) The fold increase of adriamycin IC50 value in MCF‐7 after transfected with empty vector or pcDNA3‐Flag‐GSTpi. (D–E) Western blot analysis of GSTpi protein in MCF‐7/ADR after transfected with pLKO.1 or GSTpi‐shRNA. (F) The fold change of adriamycin IC50 value in MCF‐7/ADR after transfected with pLKO.1 or GSTpi‐shRNA. (G–H) Western blot assay of GSTpi in three GSTpi knockdown MCF‐7/ADR cell lines. (I) The fold change of adriamycin IC50 value in three GSTpi knockdown MCF‐7/ADR cell lines. *p < 0.05, **p < 0.01, ***p < 0.001.

In order to investigate the effect of GSTpi on breast cancer cell sensitivity to ADR, the pcDNA3‐Flag‐GSTpi (WT) was transfected into MCF‐7. GSTpi could be detected by western blot and immunofluorescence after transfected with pcDNA3‐Flag‐GSTpi (WT), compared with the empty vector (Figure 2A, B). CCK ‐8 was employed to analyze the IC50 of the cells to ADR. As shown in Figure 2C, the IC50 of MCF‐7 in the pcDNA3‐Flag‐GSTpi (WT) transfected group was significantly increased. Besides, we constructed the pLKO.1‐GSTpi shRNA to silence GSTpi gene (Figure S1A) and pLKO.1‐GSTpi shRNA was transfected into MCF‐7/ADR. The exact sequences of shRNA were shown in Table S1. GSTpi expression was reduced by 47% in MCF‐7/ADR transfected with the pLKO.1‐GSTpi shRNA (Figure 2D, E). Downregulated GSTpi in MCF‐7/ADR significantly decreased the IC50 of ADR, compared with the pLKO.1 empty vector transfection (Figure 2F). To further demonstrate the effect of GSTpi on ADR sensitivity, pLentiCRISPRv2‐GSTpi was constructed (Figure S1B) and used to transfect MCF‐7/ADR cells by using viral packaging 293 T cells. The sequences of pLentiCRISPRv2‐GSTpi (sgRNA) were shown in Table S2. The durable concentration of MCF‐7/ADR cells to was detected and stable expression clones were screened and amplified by medium containing puromycin. As shown in Figure 2G, H, three GSTpi knockdown MCF‐7/ADR cell lines were selected, and their IC50 also decreased compared with the control group (Figure 2I).

### 3.3 Effects of NBDHEX on GSTpi enzyme activity and the combination of ADR with NBDHEX on MCF‐7/ADR cell viability and apoptosis

**FIGURE 3:**
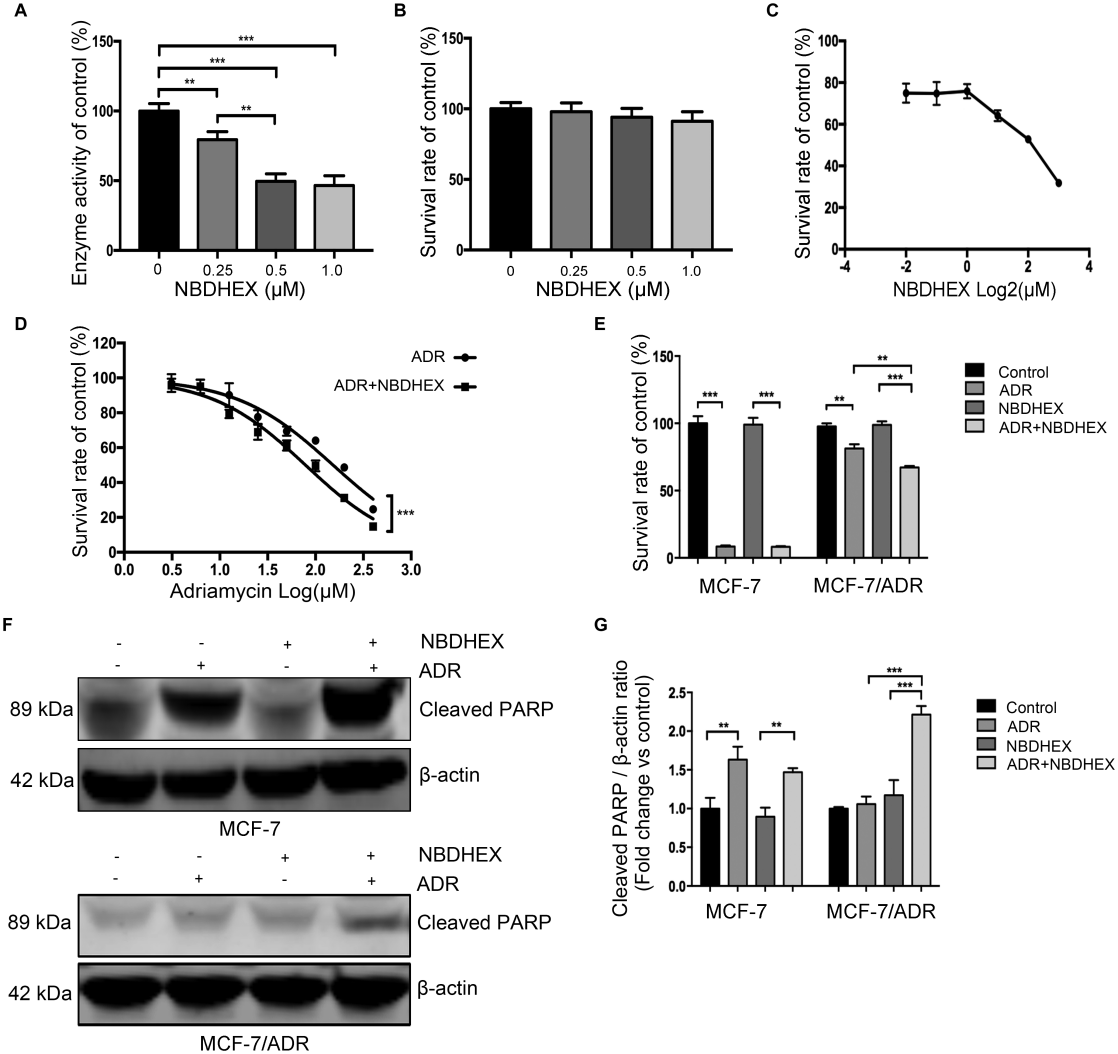
Co‐treatment of NBDHEX and adriamycin could promote the apoptosis of MCF‐7/ADR cell via inhibiting GSTpi enzyme activity. (A) GSTpi enzyme activity after treatment of NBDHEX. (B–C) Survival rate of MCF‐7/ADR cell line after NBDHEX treatment. (D) Survival curve of MCF‐7/ADR cell line after adriamycin or adriamycin+NBDHEX treatment. (E) Cell viability assays of MCF‐7 and MCF‐7/ADR after treatment of NBDHEX, adriamycin or adriamycin+NBDHEX. (F–G) Western blot assays of cleaved PARP in MCF‐7 and MCF‐7/ADR after treatment of NBDHEX, adriamycin or adriamycin+NBDHEX. β ‐actin was used as an internal control. **p < 0.01, ***p < 0.001.

The structure and validation of NBDHEX were showed previously.^23^ Purified GSTpi (WT) protein and GST activity assay kit were applied to explore the NBDHEX effect on MCF‐7/ADR cell and GSTpi enzyme activity. The results demonstrated that 0.5–1.0 μM NBDHEX could inhibit 50% of GSTpi enzymatic activity while it had no influence on MCF‐7/ADR cell viability when NBDHEX concentration was less than 1.0 μM (Figure 3A, B).

Concentration more than 1.0 μM of NBDHEX might suppress cell viability (Figure 3C). Herein, we used 0.5 μM NBDHEX to further investigate its role on GST activity in MCF‐7/ADR cell since this drug concentration had the similar effect to 1.0 μM NBDHEX on the enzymatic activity of GSTpi. Therefore, we co‐conducted NBDHEX (0.5 μM) and progressive increasing concentrations of ADR on MCF‐7/ADR cells for further analysis. The CCK‐8 assay results suggested combination of NBDHEX and ADR significantly suppressed cell viability when ADR concentration was more than 12.5 μM (Figure 3D), indicating that NBDHEX had synergistic effects on reversing the ADR‐resistance. Then, we used NBDHEX (0.5 μM) and ADR (12.5 μM) to perform CCK‐8 assay and Western blot on both MCF‐7 and MCF‐7/ADR cells. As shown in Figure 3E, treatment with ADR and NBDHEX in MCF‐7/ADR cells significantly decreased the cell viability when compared with single ADR. However, NBDHEX had no synergistic effects on MCF‐7 cell viability. Moreover, Western blot assay displayed that the expression level of cleaved PARP in the combination group was higher than the level in the ADR group. However, single ADR or co‐administration of ADR and NBDHEX had the same effect on cell apoptosis in MCF‐7 cell (Figure 3F, G). All together, these data demonstrated that co‐treatment of NBDHEX and adriamycin could promote the apoptosis of MCF‐7/ADR cells via inhibiting GSTpi enzyme activity.

### 3.4 GSTpi phosphorylation correlated with enzyme activity

**FIGURE 4:**
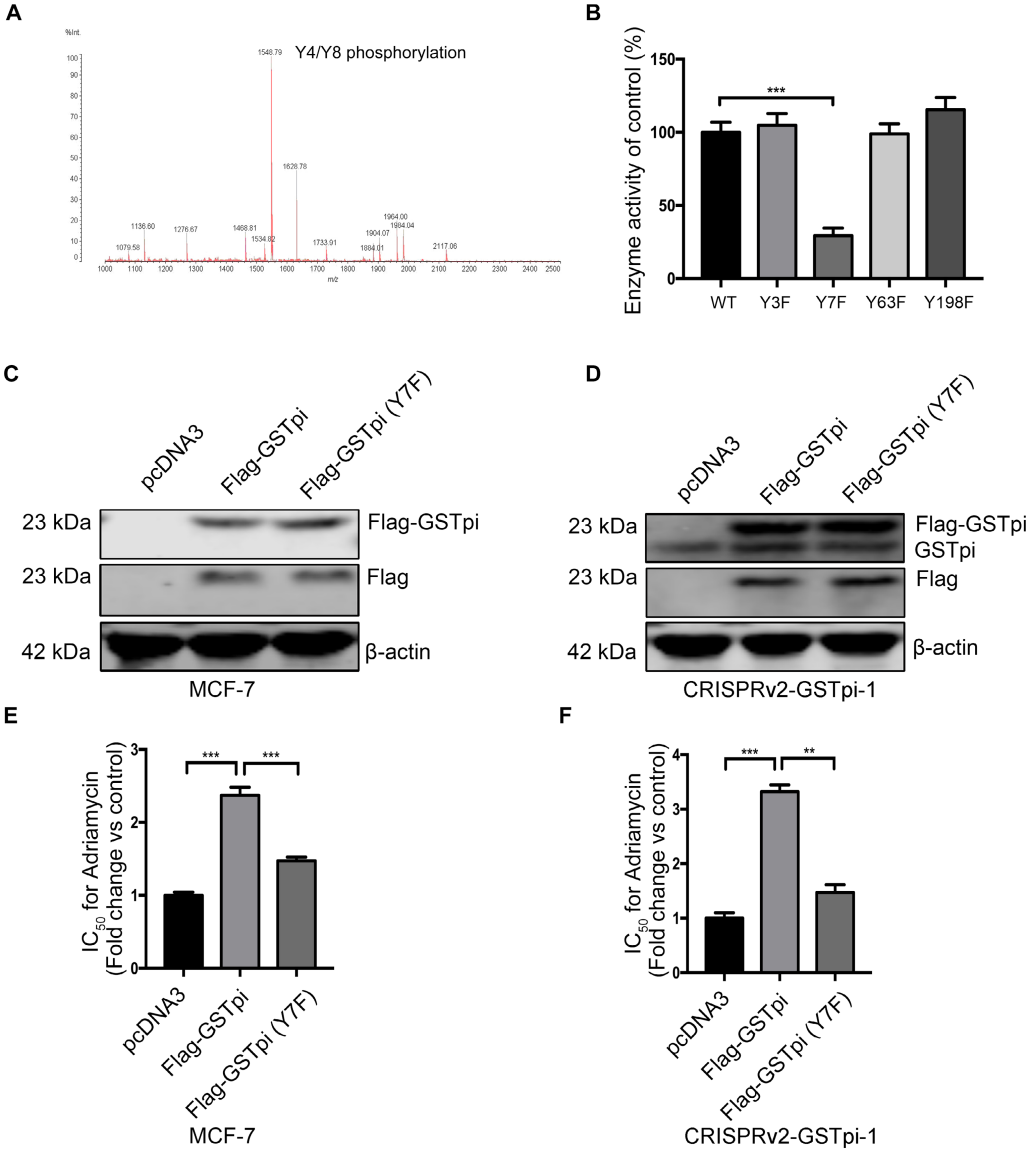
GSTpi phosphorylation determined GSTpi enzyme activity. (A) Mass spectrometry analysis of phospho‐acceptor residues in GSTpi protein in MCF‐7/ADR cell. (B) GSTpi enzyme activity of the four inactive mutant proteins. (C) Western blot assay of GSTpi in MCF‐7 after transfected with empty vector, pcDNA3‐Flag‐GSTpi or pcDNA3‐Flag‐GSTpi(Y7F). (D) Western blot assay of GSTpi in three GSTpi knockdown cell line after transfected with empty vector, pcDNA3‐Flag‐GSTpi or pcDNA3‐Flag‐GSTpi(Y7F). (E) The fold change of adriamycin IC50 value in MCF‐7/ADR after transfected with empty vector, pcDNA3‐Flag‐GSTpi or pcDNA3‐Flag‐GSTpi(Y7F). (F) The fold change of adriamycin IC50 value in GSTpi knockdown cell lines after transfected with empty vector, pcDNA3‐Flag‐GSTpi or pcDNA3‐Flag‐GSTpi(Y7F). **p < 0.01, ***p < 0.001.

Previous reports have indicated that protein phosphorylation might play an important role in GSTpi enzyme activity.^24^, ^25^ Mass spectrometry analysis was applied to further investigate the relationship between GSTpi phosphorylation and GSTpi enzyme activity. The results identified tyrosine‐3, 7, 63, 198 were phospho‐acceptor residues in GSTpi protein in MCF‐7/ADR cell (Figure 4A and Table S3). Therefore, we constructed and purified the inactive mutant proteins including GSTpi (Y3F), GSTpi (Y7F), GSTpi (Y63F), GSTpi (Y198F) (phenylalanine replaced tyrosine in the 3, 7, 63, 198 amino‐terminal position) and detected their activity using GST activity assay kit (Figure 4B, Figure S1C, D). The sequences of expression vector were shown in Table S4. The results of enzyme activity and CCK‐8 showed that phosphorylation of tyrosine‐7 in GSTpi enhanced the activity of GSTpi, and could mediate the resistance of both the parental and ADR‐resistant breast cancer cells to ADR (Figure 4C–F).

### 3.5 GSTpi phosphorylation promoted GSTpi nuclear translocation

**FIGURE 5:**
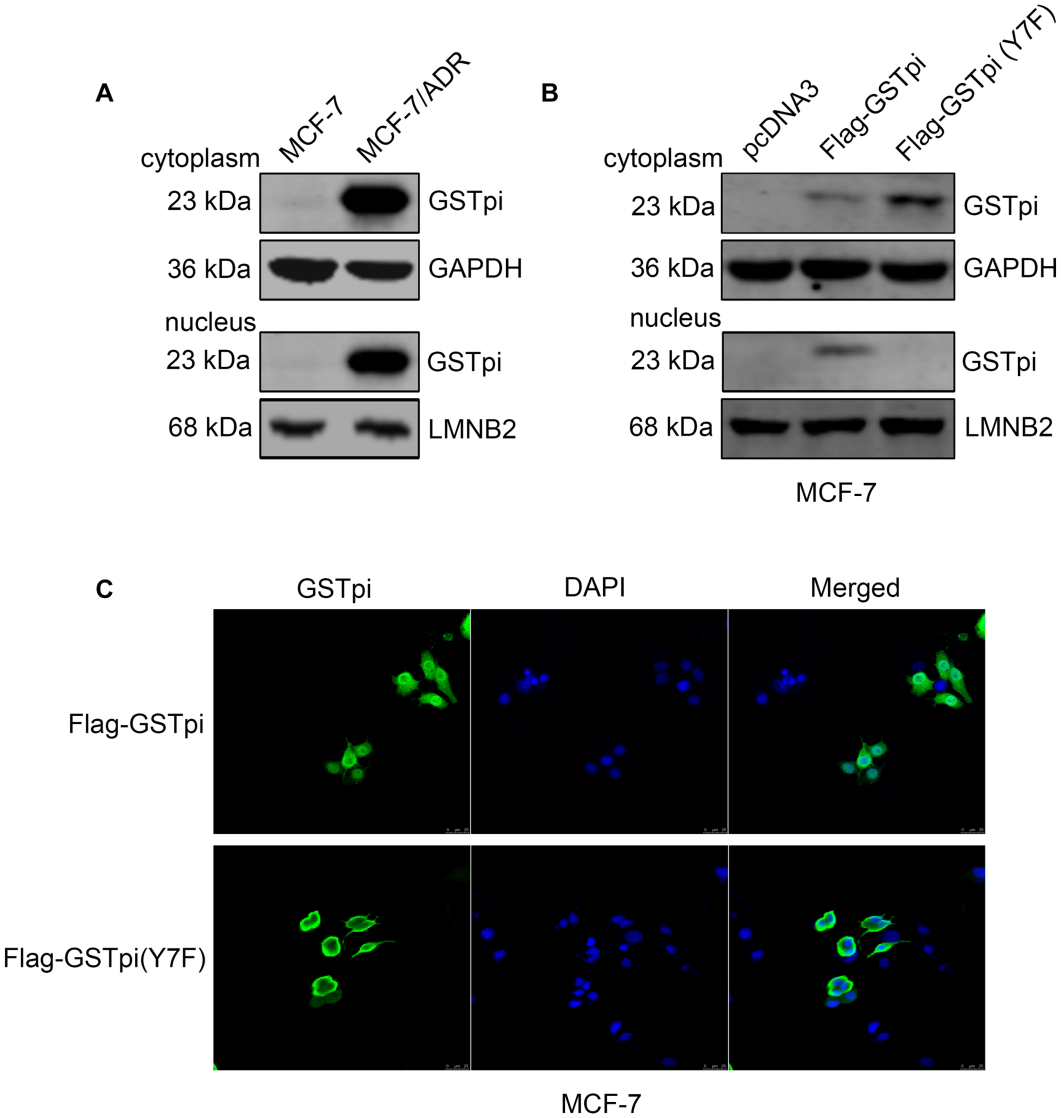
GSTpi phosphorylation promoted GSTpi nuclear translocation. (A) Western blot assay of GSTpi in cytoplasm and nucleus of MCF‐7 and MCF‐7/ADR. (B) Western blot assay of GSTpi in cytoplasm and nucleus of MCF‐7 after transfected with empty vector, pcDNA3‐Flag‐GSTpi or pcDNA3‐Flag‐GSTpi(Y7F). (C) The immunofluorescence scanning of GSTpi in cytoplasm and nucleus of MCF‐7 after transfected with empty vector, pcDNA3‐Flag‐GSTpi or pcDNA3‐Flag‐GSTpi(Y7F). Scale bars represent 25 μm.

Interestingly, from Figure 1E, wo observed that GSTpi not only was high‐expressed in MCF‐7/ADR but also distributed in the nucleus of the cancer cell. Cytoplasmic and nuclear proteins were prepared and the Western blot assay verified its intracellular location (Figure 5A). Then, we transfected pcDNA3, pcDNA3‐Flag‐GSTpi (WT) and pcDNA3‐Flag‐GSTpi (Y7F) in MCF‐7 to study the correlation between GSTpi phosphorylation and intracellular location. Both results of Western blot and immunofluorescence displayed that GSTpi was detected in cytoplasm and nuclear in pcDNA3‐Flag‐GSTpi (WT) group while was only observed in cytoplasm in pcDNA3‐Flag‐GSTpi (Y7F) group (Figure 5B, C). These data noted that, in addition to influencing its enzyme activity, tyrosine‐7 phosphorylation in GSTpi might promote GSTpi nuclear translocation as well. However, we could not directly confirm the serendipitous discoveries. Since there were few reports about the function of GSTpi in the nucleus, the relationship between adriamycin resistance and GSTpi location warranted further study.

### 3.6 In vivo studies

**FIGURE 6:**
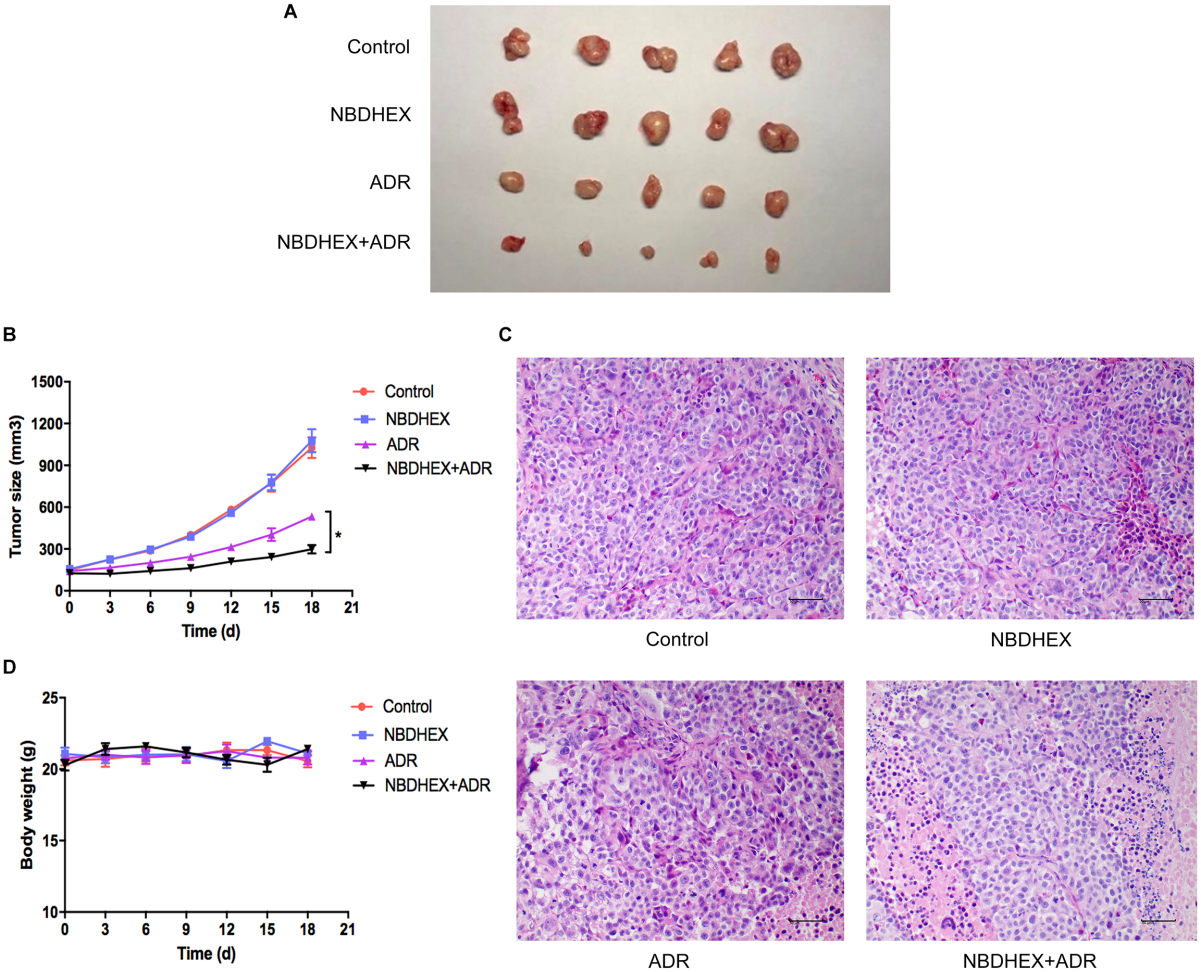
NBDHEX increases ADR efficacy against breast cancer in vivo. (A) Collection of tumors after removal from mice (5/group). (B) The tumor volumes were calculated every 3 days after inoculation. (C) Hematoxylin–eosin staining was performed of tumor samples collected. Scale bars represent 50 μm. (D) The mice weights were measured every 3 days after inoculation. *p < 0.05.

The efficacy of ADR or NBDHEX as monotherapy and the two‐agent combination was investigated. Compared with control group, NBDHEX monotherapy did not induced tumor growth inhibition (Figure 6A, B). Noteworthy, two‐agent combination significantly increased tumor growth reduction in comparison with single agent treatment (Figure 6A, B). At sacrifice, cancer nodules were excised and histological examination revealed a reduction of tumor infiltration in the two‐agent combination group (Figure 6C). Besides, the combination therapy was well tolerated in the experiment (Figure 6D).

## 4 DISCUSSION

ADR is one of the most effective chemotherapeutic agents for breast cancer treatment. Nevertheless, Drug resistance severely limits its clinical efficacy, which was an urgent problem to be resolved. In this work, we tackled the efficacy of NBDHEX on the ADR‐resistance breast cancer cell and focused attention on GSTpi enzymatic activity and its own phosphorylation. Many evidence demonstrated that GSTpi overexpression caused resistance of cancer cells to assorted anticancer drugs and MCF‐7 was a GSTpi‐absent cell line.^6^, ^8^, ^22^, ^26^ Therefore, we used MCF‐7 as a negative control cell line and we constructed the ADR‐resistant cell line of MCF‐7 to detect GSTpi expression in the two cell lines firstly. GSTpi was high expressed and distributed in nuclei in MCF‐7/ADR while was absent in MCF‐7. In addition, results based on pLentiCRISPRv2‐GSTpi experiments and IHC staining in breast cancer tissues further confirmed the relationship between GSTpi expression and ADR resistance. Moreover, Flag‐GSTpi and shRNA‐GSTpi were used to verified these findings, which were also confirmed by a previous study.^27^

Taking into account its important role in cancers, different inhibitors targeting GSTpi have been reported.^15^, ^28^, ^29^ Among them, we noticed that NBDHEX, which could significantly suppress GSTpi activity was emphasized as a potential anticancer drug in recent years.^19^, ^20^, ^30^, ^31^ We reviewed previous study and understandings of NBDHEX, however, its role in ADR‐resistance breast cancer cell remained unexplored.^21^ Tentori et al. showed that NBDHEX increased temozolomide efficacy against malignant melanoma.^32^ We then explored whether NBDHEX could increase ADR efficacy in breast cancer cells. In this study, 0.5–1.0 μM NBDHEX inhibited 50% activity of GSTpi, which was consistent with a previous study^16^ though drug concentration in this range did not significantly inhibit the viability of MCF‐7/ADR cell. We used 0.5 μM NBDHEX to perform further assays when we compared NBDHEX and ADR combination with single ADR, so as to eliminate the influence of single NBDHEX on cell viability. The results indicated that 0.5 μM NBDHEX could significantly reduce the resistance of MCF‐7/ADR to ADR. However, a previous study reported that inhibition of GST through NBDHEX could slightly overcome the resistance of ADR. In that study, the concentration of ADR used was 10 μM,^27^ and the CCK‐8 assay results in our study suggested NBDHEX with ADR suppressed cell viability when ADR concentration was more than 12.5 μM. We considered that the concentration of ADR and the resistance degree of different MCF‐7/ADR cell lines might affect the outcomes although the same cell model was used.

Although flow cytometry assay could not be carried out perfectly because the red color of ADR interfered strongly in the process of flow cytometric staining (Data not shown). The Western blot assay for cleaved PARP confirmed that NBDHEX combined with ADR could promote apoptosis in comparison with single ADR. In MCF‐7 cell, co‐administration of ADR and NBDHEX did not increase ADR efficacy, mainly because it is a GSTpi‐ absent cell and low concentration of NBDHEX did not affect the proliferation and apoptosis of the cell. Furthermore, in vivo study, NBDHEX and ADR combination significantly inhibited tumor growth when compared with ADR monotherapy. These results indicated that NBDHEX increased ADR efficacy against breast cancer by inhibiting the activity of GSTpi. Some previous studies demonstrated compounds targeting GSTpi increased the levels of cleaved PARP.^33^, ^34^ However, NBDHEX did not directly act on DNA in our study because the PARP expression level in the control group is similar with the level in the NBDHEX treatment group. In fact, this compound in combination with ADR provoked synergistic anti‐proliferative effects against breast cancer cells.

The most widely studied function of GSTs is the conjugation reaction of reduced GSH to endogenous and xenobiotic compounds. GSTs can catalyze the binding of GSH and many anticancer drugs, which are substrates for GSTs and extruded from cells efficiently.^35^, ^36^, ^37^ In some cancers, the detoxifying activity of GSTs still play an important role in drug resistance via activating the GST/GSH cellular system.^6^, ^35^ Additionally, enhance GSTP1 gene expression or protein level, or both seem to be related with increased GSTpi enzymatic activity. Moreover, both enzymatic activity and intracellular levels of GSTPi might associated with the degree of cisplatin resistance.^22^ On the basis of these findings, ADR might be a substrate for GSTpi/GSH system. However, more evidence is required to assess whether NBDHEX can hinder conjugation of ADR to GSH mediated by GSTpi and increase intracellular accumulation of ADR.

Some studies suggested that the phosphorylation of serine, threonine and tyrosine on GSTpi could enhance the activity of GSTpi.^24^, ^25^, ^38^ Therefore, we utilized mass spectrometry to detect the phosphorylation sites of GSTpi in MCF‐7/ADR. Four tyrosine residues including 3, 7, 63 and 198 sites were found to be phosphorylated and the activity of GSTpi was mainly dependent on tyrosine‐7 phosphorylation. Remarkably, a reduction of MCF‐7/ADR resistance to ADR was observed when tyrosine‐7 phosphorylation was inactivated, indicating that tyrosine‐7 phosphorylation of GSTpi might mediate breast cancer resistance.

In the process of immunofluorescence detection of GSTpi expression in drug‐resistant cell, we found that GSTpi was mainly distributed in the nucleus. Subsequent Western blot assay further confirmed that GSTpi was highly expressed in MCF‐7/ADR nuclei. We then investigated the relationship between GSTpi phosphorylation and its intracellular localization. The results showed that GSTpi was mainly located in cell cytoplasm after inhibiting phosphorylation at tyrosine‐7 of GSTpi, which indicated that phosphorylation at tyrosine‐7 might be related to GSTpi nuclear translocation.

There are a number of important strengths and weaknesses to our study. First, ADR might be a substrate for GSTpi/GSH system. However, more evidence is required to confirm the conjecture. Next, our result indicated that NBDHEX increased ADR efficacy by inhibiting the activity of GSTpi. and tyrosine‐7 phosphorylation of GSTpi might mediated breast cancer resistance to ADR. However, due to the lack of commercial tyrosine‐7 phosphorylation antibody of GSTpi, we could not directly confirm the relationship between NBDHEX and tyrosine‐7 phosphorylation of GSTpi. Third, it is showed that phosphorylation at tyrosine‐7 might be related to GSTpi nuclear translocation. Since there were few reports about the function of GSTpi in the nucleus, the relationship between adriamycin resistance and GSTpi location warranted further study.

## 5 CONCLUSIONS

In summary, this study demonstrated NBDHEX was capable of enhancing the antitumor activity of ADR in vitro and in vivo. All these observations indicated NBDHEX as a good candidate for combination with ADR for breast cancer treatment via inhibiting GSTpi activity.

## AUTHOR CONTRIBUTIONS

**Huanhuan Sha:** Formal analysis (lead); methodology (lead); resources (lead); writing – original draft (lead); writing – review and editing (lead). **Renrui Zou:** Formal analysis (equal); methodology (equal); resources (equal); writing – review and editing (equal). **Ya Lu:** Formal analysis (supporting); investigation (supporting); methodology (supporting); resources (supporting); writing – review and editing (supporting). **Yujie Gan:** Methodology (supporting); resources (supporting); writing – review and editing (supporting). **Rong Ma:** Supervision (equal); validation (equal). **Jifeng Feng:** Conceptualization (lead); funding acquisition (lead); project administration (lead); supervision (equal); validation (equal). **Dan Chen:** Conceptualization (equal); funding acquisition (equal); project administration (equal); resources (equal); supervision (equal); validation (equal).

## FUNDING INFORMATION

This study was supported by the Jiangsu Provincial Medical Youth Talent (QNRC2016653), the National Natural Science Foundation of China (grant no. 81602441), and Jiangsu Provincial Key Research Development Program (grant no. BE2016794).

## CONFLICT OF INTEREST

The authors declare that they have no conflicts of interest.

## ETHICS APPROVAL

The in vivo study was approved by Ethic Committee of the Nanjing Medical University. The use of already available data and formalin‐fixed, paraffin‐embedded sections in this study was approved by Ethic Committee of the Affiliated Cancer Hospital of Nanjing Medical University. Written informed consent was obtained from patients for use of tissues.

## Supporting information


Figure S1

Table S1

Table S2

Table S3

Table S4

## Data Availability

The data generated and/or analyzed during the study are available from the corresponding author on reasonable request.
