# Supplementary figures and images for: NBDHEX re‐sensitizes adriamycin‐resistant breast cancer by inhibiting glutathione S‐transferase pi
Source: Cancer Med. 2022 Oct 20;12(5):5833–45. doi: 10.1002/cam4.5370 (PMC10028113; doi:10.1002/cam4.5370)

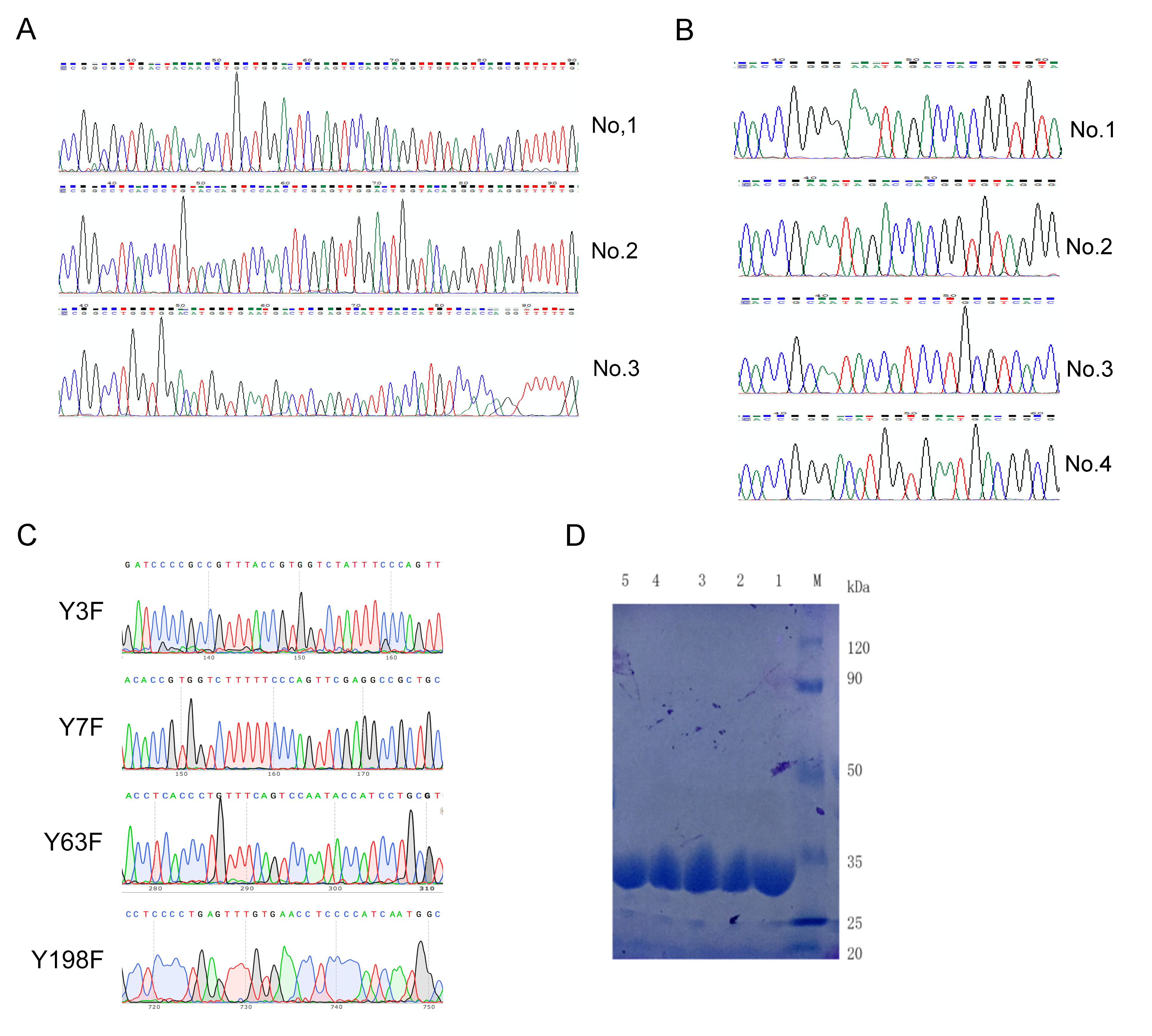

Supplement: Supplementary file 1 — Figure S1 [file CAM4-12-5833-s003.tif]
