# Supplementary material for: NBDHEX re‐sensitizes adriamycin‐resistant breast cancer by inhibiting glutathione S‐transferase pi
Source: Cancer Med. 2022 Oct 20;12(5):5833–45. doi: 10.1002/cam4.5370 (PMC10028113; doi:10.1002/cam4.5370)
Supplement: Supplementary file 2 — Table S1 [file CAM4-12-5833-s002.docx]

Supplementary Table 1.

Sequences of the primers sets for silencing GSTpi gene.

| Primer | Direction | Nucleotide sequence (5’–3’) |
| --- | --- | --- |
| No.1 | Forward | CCGGCGCTGACTACAACCTGCTGGACTCGAGTCCAGCAGGTTGTAGTCAGCGTTTTTG |
| No.1 | Reverse | AATTCAAAAACGCTGACTACAACCTGCTGGACTCGAGTCCAGCAGGTTGTAGTCAGCG |
| No.2 | Forward | CCGGCCTCACCCTGTACCAGTCCAACTCGAGTTGGACTGGTACAGGGTGAGGTTTTTG |
| No.2 | Reverse | AATTCAAAAACCTCACCCTGTACCAGTCCAACTCGAGTTGGACTGGTACAGGGTGAGG |
| No.3 | Forward | CCGGCCTGGTGGACATGGTGAATGACTCGAGTCATTCACCATGTCCACCAGGTTTTTG |
| No.3 | Reverse | AATTCAAAAACCTGGTGGACATGGTGAATGACTCGAGTCATTCACCATGTCCACCAGG |
