# Supplementary material for: NBDHEX re‐sensitizes adriamycin‐resistant breast cancer by inhibiting glutathione S‐transferase pi
Source: Cancer Med. 2022 Oct 20;12(5):5833–45. doi: 10.1002/cam4.5370 (PMC10028113; doi:10.1002/cam4.5370)
Supplement: Supplementary file 3 — Table S2 [file CAM4-12-5833-s004.docx]

Supplementary Table 2.

Sequences of the primers sets for pLentiCRISPRv2-GSTpi.

| Primer | Direction | Nucleotide sequence (5’–3’) |
| --- | --- | --- |
| No.1 | Forward | CACCGGGGAAATAGACCACGGTGTA |
| No.1 | Reverse | AAACTACACCGTGGTCTATTTCCCC |
| No.2 | Forward | CACCGAAATAGACCACGGTGTAGGG |
| No.2 | Reverse | AAACCCCTACACCGTGGTCTATTTC |
| No.3 | Forward | CACCGCAATACCATCCTGCGTCACC |
| No.3  No.4  No.4 | Reverse  Forward  Reverse | AAACGGTGACGCAGGATGGTATTGC  CACCGGGACATGGTGAATGACGGCG  AAACCGCCGTCATTCACCATGTCCC |
