# Supplementary material for: NBDHEX re‐sensitizes adriamycin‐resistant breast cancer by inhibiting glutathione S‐transferase pi
Source: Cancer Med. 2022 Oct 20;12(5):5833–45. doi: 10.1002/cam4.5370 (PMC10028113; doi:10.1002/cam4.5370)
Supplement: Supplementary file 4 — Table S3 [file CAM4-12-5833-s001.docx]

Supplementary Table 3

Mass spectrometry analysis of phospho-acceptor residues in GSTpi protein in MCF-7/ADR cell

| Source: | GST Pi_ Homo |  |  |  |  |
| --- | --- | --- | --- | --- | --- |
| Peptide sequence | Mr (expt) | Mr (calc) | Position | Modifications | Mascot ion score |
| ASCLYGQLPK | 1079.58 | 1079.56 | 46-55 | C48 carbamidomethyl | 13 |
| AScamCLYGQLPK | 1136.60 | 1136.58 | 46-55 |  | 14 |
| MLLADQGQSWK | 1276.67 | 1276.64 | 20-30 |  | 14 |
| MPPYTVVYFPVR | 1468.81 | 1468.77 | 1-12 |  | 17 |
| YISLIYTNYEAGK | 1534.82 | 1534.78 | 104-116 |  | 12 |
| MPPpYTVVYFPVR | 1548.79 | 1548.76 | 1-12 | Y4/Y8 phosphorylation | 25 |
| MPPpYTVVpYFPVR | 1628.78 | 1628.75 | 1-12 | Y4+Y8 phosphorylation | 21 |
| EEVVTVETWQEGSLK | 1733.91 | 1733.86 | 31-45 |  | 11 |
| FQDGDLTLYQSNTILR | 1884.01 | 1883.95 | 56-71 |  | 13 |
| AFLASPEYVNLPINGNGK | 1904.07 | 1903.99 | 192-209 |  | 16 |
| FQDGDLTLpYQSNTILR | 1964.00 | 1963.93 | 56-71 | Y64 phosphorylation | 18 |
| AFLASPEpYVNLPINGNGK | 1984.04 | 1983.98 | 192-209 | Y199 phosphorylation | 15 |
| DQQEAALVDMVNDGVEDLR | 2117.06 | 2116.98 | 83-101 |  | 16 |
