# Supplementary material for: NBDHEX re‐sensitizes adriamycin‐resistant breast cancer by inhibiting glutathione S‐transferase pi
Source: Cancer Med. 2022 Oct 20;12(5):5833–45. doi: 10.1002/cam4.5370 (PMC10028113; doi:10.1002/cam4.5370)
Supplement: Supplementary file 5 — Table S4 [file CAM4-12-5833-s005.docx]

Supplementary Table 4.

Sequences of the primers sets for four mutants of GSTpi.

| Primer | Direction | Nucleotide sequence (5’–3’) |
| --- | --- | --- |
| Y3F | Forward | ATCCCCGCCGTTTACCGTGGTCTATTTCCCAGT |
| Y3F | Reverse | AGACCACGGTAAACGGCGGGGATCCGCGACCCA |
| Y7F | Forward | CACCGTGGTCTTTTTCCCAGTTCGAGGCCGCTG |
| Y7F | Reverse | GAACTGGGAAAAAGACCACGGTGTACGGCGGGG |
| Y63F | Forward | CCTCACCCTGTTTCAGTCCAATACCATCCTGCG |
| Y63F  Y198F  Y198F | Reverse  Forward  Reverse | TATTGGACTGAAACAGGGTGAGGTCTCCGTCCT  CTCCCCTGAGTTTGTGAACCTCCCCATCAATGG  GGAGGTTCACAAACTCAGGGGAGGCCAGGAAGG |
